# Ultrasound-guided approach to surgery for nodal recurrence following lateral neck dissection for differentiated thyroid carcinoma. A single institution experience

**DOI:** 10.3389/fsurg.2024.1403741

**Published:** 2024-06-25

**Authors:** Mario Pacilli, Giovanna Pavone, Andrea Quazzico, Alberto Fersini, Antonio Ambrosi, Nicola Tartaglia

**Affiliations:** ^1^ Department of Medical and Surgical Sciences, University of Foggia, Foggia, Italy; ^2^ Hospital “Mons. Dimiccoli”, Barletta, Italy

**Keywords:** thyroid carcinoma, nodal recurrences, intraoperative ultrasound, lateral neck dissection, neck surgery

## Abstract

**Introduction:**

Total thyroidectomy (TT) and central neck dissection (CND) had a significant effect on the reduction of local recurrence compared with TT alone. Lateral Neck Dissection (LND) was performed in all the cases with therapeutic intent. The suspicion of nodal recurrence is provided by the appearance of one or more enlarged nodes in the central and/or laterocervical compartment during the follow up period.

**Methods:**

From January 2018 to November 2023, 16 patients at the University General Surgery unit of the Polyclinic of Foggia underwent reoperation due to nodal recurrence after previously undergoing total thyroidectomy with central and lateral cervical dissection.

**Results:**

All surgical interventions were approached with intraoperative ultrasound performed by the operating surgeon. In all cases, ultrasound identification of the suspicious lymph node led to histological confirmation of malignancy. In only two cases it was necessary to carry out an extemporaneous intraoperative histological examination. No complications were recorded during the operations.

**Conclusions:**

Surgical reintervention in patients with nodal recurrence is challenging and requires an assessment by members of the interdisciplinary team. The ideal method should be economically convenient, easy to practice, with a quick learning curve, easily reproducible, and safe for patients. Intraoperative, ultrasound-guided, is a safe and effective technique. It facilitates tumor localization and removal, especially in patients requiring re-operative neck surgery.

## Introduction

Differentiated thyroid carcinoma (DTC) is a gradually increasing tumor with an excellent prognosis and a 10-year survival rate above 90% (1). Data from 2006 to 2012 indicate that the annual incidence of thyroid cancer is approximately 5.4% in men and 6.5% in women. In addition, thyroid cancer is the fifth most common malignant tumor in women and the most common cancer in women under the age of 25, with an average annual incidence rate of 6.2% (2, 3). Surgery is certainly still the cornerstone in treatment and implicates the removal of the thyroid with associated removal of the involved cervical lymph nodes.

Nodal involvement may include the central compartment (CC) or the lateral cervical compartment (LC), and the lateral neck dissection (LND) is considered an important surgical procedure and is executed merely with therapeutic intent when node involvement is confirmed (preoperatively or intraoperatively) and not for prophylactic purpose as in the case of central neck dissection (CND) (4). However, nodal recurrent disease (defined as clinically detectable disease in the central and/or lateral compartment lymph nodes) (5), it is the most frequent site of disease recurrence, although it is by no means a very frequent event, especially in cases in which a lymph node dissection has already been performed (6).

Surgery for lateral neck lymph node recurrence following LND represents a challenging event for both patients and surgeons due to the difficulties of reoperation, including an increasing risk of postoperative morbidities.

The aim of this study is to demonstrate how in our center, the use of intraoperative ultrasound can allow surgery to be conducted in a safe and effective manner.

## Methods

Between January 2018 and November 2023, nearly 400 thyroid surgeries were performed at the University General Surgery unit of the Polyclinic of Foggia, addressing both benign and malignant conditions, including central and lateral neck dissections.

This study included patients who had previously undergone total thyroidectomy with central neck dissection (CND) and lateral neck dissection (LND) and who experienced nodal recurrence during the follow-up period, presenting a significant surgical challenge. Patients without evidence of nodal recurrence or those with locoregional or distant recurrence were excluded from the study.

Postoperative follow-up was conducted for all patients, involving consistent hormonal suppressive treatment. Patients underwent an ultrasound neck evaluation initially six months post-surgery, followed by annual assessments, including the evaluation of thyroglobulin (Tg) levels. Radioactive iodine (RAI) therapy was considered based on the stage and associated risk factors. In cases of suspected nodal recurrence, fine-needle aspiration cytology (FNAC) and scintigraphic studies were performed for further evaluation.

## Results

Overall 16 patients were included in this study: 5 male and 11 female and a mean age of 55.25 ± 11.44 years. All preoperative patients' characteristics are reported in Table 1.

**Table 1:** Preoperative patients’ characteristics.

*N* patients	16
Age (years)	
Range	38–72
Mean	55.25
Median	55.5
M:F	11:5
pT stage	
T1/T2	6
T3/T4	10
Multifocality Yes/No	12/4
Extracapsular invasion	14/2
No. of removed lateral neck nodes	
Range	22–46
Mean	35.93
Median	34.5
No. of metastatic lateral neck nodes	
Range	4–8
Mean	5.81
Median	6
Lymph node ratio	
Range	0.17–0.22
Mean	0.18
Median	0.2
Bilateral lateral neck dissection Yes/No	4/12
Comprehensive LND (levels II–V)/Limited LND	11/5
First surgery outside our Institution Yes/No	9/7

All patients previously underwent total thyroidectomy + CND + LND. The postoperative histological examination showed a prevalence of pT3/T4 cases, multifocality and extracapsular invasion. The average number of lymph nodes removed was 35.93, with an average lymph node ratio of 0.18. Bilateral lymphadenectomy was performed in 4 patients, and 7 operations were conducted at a center other than ours.

All patients examined in this study developed nodal recurrence (Table 2). Nodal recurrence was diagnosed during the follow-up period, with an average time of 6.21 years. The patients underwent a complete neck ultrasound, followed by FNAC or scintigraphic examination. In all cases, lateral neck lymph node recurrence was shown on the ultrasound and confirmed with FNAC, except for one case where the position and relationship of the suspicious lymph node with the large vessels of the neck made the procedure impractical and unsafe.

**Table 2:** Data regarding nodal recurrence.

Nodal recurrence	16/16
Time of recurrence (years)	
Range	2–8.2
Mean	6.21
Median	6.20
DS	±1.29
Diagnostic tools	
- FNAC Yes/No	15/1
- Scintigraphy Yes/No	14/2
Previous RAI treatment	16/16
Ultrasound-guided surgical approach	16/16
Correspondence with preoperative ultrasound Yes/No	11/5
Need for extemporaneous histological examination Yes/No	2/14
Postoperative complication	//
Postoperative hospital stay (days)	
Range	1–2
Mean	1.19
Median	1.00

**Figure 1:**
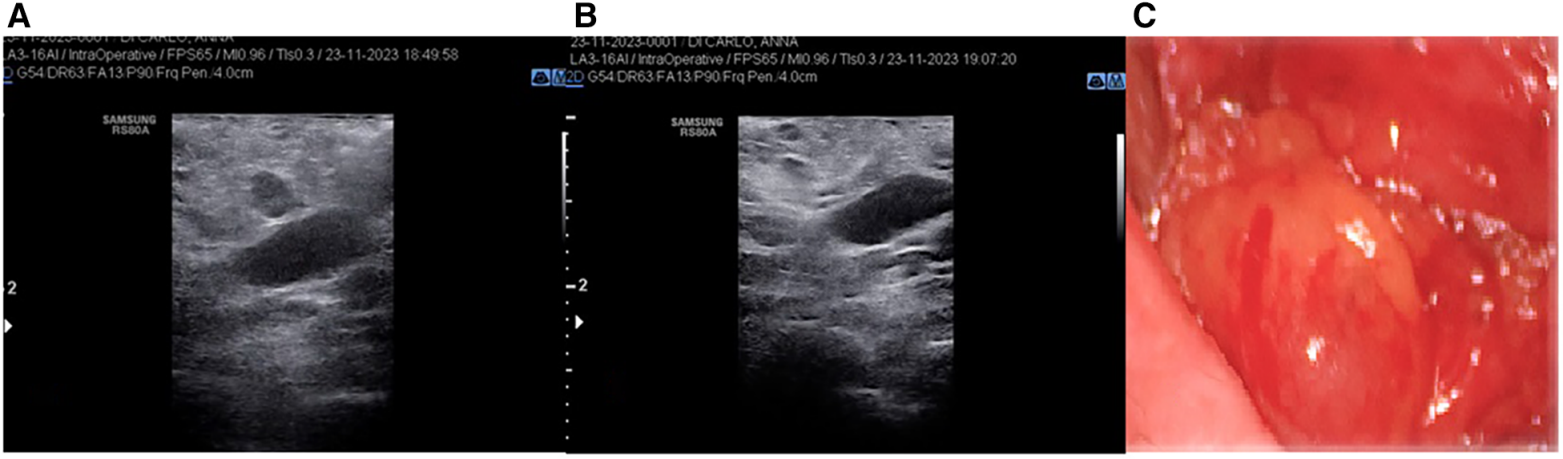
(**A**) Intraoperative ultrasound detection of suspicion node; (**B**) intraoperative ultrasound control after node's removal (**C**) pathological node.

In all cases, preoperative and intraoperative ultrasounds were performed by the surgical team. Knowing the exact location of the suspected lymphadenopathy is crucial for ensuring the surgical intervention is quick, effective, and safe. Therefore, in all cases of reoperation, a new ultrasound is performed with the patient already positioned on the operating bed, in addition to analyzing the preoperative ultrasound. The entire operation is conducted with the aid of an intraoperative ultrasound to ensure the correct identification and subsequent removal of the previously described lesion (Figure 1).

The value of intraoperative ultrasound is highlighted by the inconsistencies we observed between the ultrasound descriptions during follow-up and the actual findings in some cases.

Furthermore, in two cases, a frozen section examination was necessary before concluding the operation due to discrepancies between the preoperative and intraoperative ultrasound evaluations. In both instances, the diagnosis of thyroid cancer metastasis was confirmed. No postoperative complications were recorded, and the average postoperative hospital stay was 1.19 days.

## Discussion

Recurrence of DTC commonly occurs in the lymph nodes of the neck after initial treatment (7). However, extensive extension beyond the thyroid observed during surgery is also a significant factor for recurrence in the thyroid bed. When small, suspicious sub-centimeter lymph nodes are detected on ultrasound, a watch-and-wait approach can be adopted to monitor for any progression. A recent retrospective study (8) observed a 51-month progression-free survival (PFS) rate of 35% in 14 out of 40 patients who had metastatic lymph nodes measuring less than 2 cm in their largest diameter. According to the guidelines provided by the ATA, central compartment lesions larger than 5 mm (in the smallest diameter) and lateral neck lesions larger than 8 mm (in the smallest diameter) may be recommended for fine-needle aspiration (FNA) biopsy, along with Tg levels determination through aspiration if necessary, but only if a specific treatment plan is in place (9, 10).

There are some alternative treatments to surgery, such as or limited lesions (less than 2 cm) that show RAI uptake, 131I can serve as an alternative treatment, or other treatments that have been used for cases of isolated metastasis include ethanol injection and US-guided percutaneous ablation (radiofrequency, laser, microwave, or cryotherapy), but surgery remains the first choice in all cases of resectable disease (11, 12).

If a previous LND has not been performed, compartmental dissection of the target level is indicated. However, if the recurrence occurs on a neck in which lymph node dissection has already been performed, the operation may be more challenging selective excision of the lymph nodes can be made (13).

Utilizing an ultrasound-guided approach to surgery for lymph node recurrence enables targeted surgery, focused on removing a small lesion within an already manipulated surgical field. Without ultrasound guidance, there is a risk of failing to identify the suspicious lymph node, necessitating larger skin incisions to expose the surgical field adequately. This could result in longer operating times and increased morbidity for the patients.

The ultrasound performed by the operating surgeon, with the patient already positioned on the operating bed, allows the identification of ultrasound landmarks, which act as a guide for surgery and can be reproduced during surgery with intraoperative ultrasound. Further tips and tricks include measuring the size of the suspected lymph node, the distance from the skin margin, studying its shape, margins and relationships with the closest anatomical structures. If these ultrasound parameters were confirmed during surgery then identification is easier and more effective.

It's essential to acknowledge significant limitations in this study. Firstly, it's a retrospective, single-institution study. We recognize that the number of patients included is insufficient for drawing conclusions with high scientific certainty. However, the limited case count is attributed to the rarity of nodal recurrence in patients who have undergone both CND and LND. The primary goal of the study is to share our experiences and highlight the challenges encountered during reoperation for identifying suspicious lymph nodes. We propose that intraoperative ultrasound can serve as a valid, safe, effective, and cost-efficient tool in such cases.

A notable strength of this study is its novelty; there are currently no other studies in the literature describing this technique and approach. Therefore, this work represents the outcomes of a treatment method consistently applied at a single institution by the same surgical team.

## Conclusions

The most prevalent form of recurrence in DTC is the reappearance or persistent presence of lymph node disease. Managing patients with suspected recurrent or persistent nodal DTC is challenging and relies on ultrasound imaging, Tg levels, a comprehensive whole-body iodine scan, and final decisions made by an interdisciplinary team.

When clear evidence of a structural abnormality is present, surgical intervention is the optimal treatment for nodal recurrence. The goal of surgery should be curative, and the approach should be economically and logistically feasible, relatively easy to perform with a quick learning curve, and should ensure minimal interobserver variation in identifying pathological nodes, all while being safe for patients.

The intraoperative, ultrasound-guided technique is a safe and effective method. It facilitates the localization and removal of tumors, especially in patients who have already undergone neck surgery, allowing reoperation to be performed safely and effectively.

## Data Availability

The raw data supporting the conclusions of this article will be made available by the authors, without undue reservation.
